# “H” is not for hydroxychloroquine—“H” is for heparin: lack of efficacy of hydroxychloroquine and the role of heparin in COVID-19—preliminary data of a prospective and interventional study from Brazil

**DOI:** 10.1186/s12879-022-07110-1

**Published:** 2022-02-04

**Authors:** Renata de Oliveira Costa, Joyce Santos Nascimento, Cadiele Oliana Reichert, Adriana Pedroso Augusto da Costa, Maria Aparecida Pedrosa dos Santos, Alberto Macedo Soares, Carlos Eduardo Mendonça Tomé, Ricardo Leite Hayden, Cassiano Waldanski dos Santos, Bruno Barreiro, Amer Abdul Basset El-Khatib, Luís Alberto de Pádua Covas Lage, Juliana Pereira, Mônica Mazzurana Benetti

**Affiliations:** 1 Faculdade de Ciências Médicas de Santos – Centro Universitário Lusíada (FCMS, UNILUS), Santos, São Paulo, Brazil; 2 Hospital Estadual Guilherme Álvaro (HGA), Santos, São Paulo, Brazil; 3 Laboratory of Immunology and Histocompatibility (LIM-19), University of São Paulo (USP), São Paulo, Brazil; 4 Department of Hematology, Hemotherapy & Cell Therapy, University of São Paulo (USP), Av. Dr. Enéas de Carvalho Aguiar, 155 – 1st. Floor. Room 61, SP São Paulo, Brazil; 5 Laboratory of Medical Investigation in Pathogenesis and Directed Therapy in Onco-Immuno-Hematology (LIM-31), University of São Paulo (USP), Av. Dr. Enéas de Carvalho Aguiar, 155 – 1st. Floor. Room 61, São Paulo, SP Brazil

**Keywords:** COVID-19, Heparin, Hydroxychloroquine, Prognostic factors, Outcomes

## Abstract

**Background:**

COVID-19 pandemic is the major public health problem in the world actually. It’s associated with high morbidity and mortality. To date, no therapeutic measure has a curative potential. Hydroxychloroquine (HCQ) is a drug with immunomodulatory properties that has demonstrated antiviral efficacy in in vitro experiments, with conflicting results in in vivo studies.

**Methods:**

A single-center, prospective and interventional study, that evaluates the impact on mortality of the HCQ use in 154 patients hospitalized with COVID-19 in a Brazilian public hospital. The study also aims to determine prognostic factors that predict mortality, ICU admission and endotracheal intubation in this population.

**Results:**

154 patients diagnosed with COVID-19 confirmed by RT-PCR and hospitalized were included. There was a male predominance (87/154, 56.5%), median age 60 years and 88% (136/154) had comorbidities. Among these, 76% (117/154) were admitted to the ICU and 29.2% (45/154) experienced EOT. The OMR was 51.3% (79/154). There was no difference in mortality between patients treated with HCQ (*N* = 95) and non-HCQ (*N* = 59) (44.1% × 55.8%, *p* = 0.758). In univariate analysis, age ≥ 60 years (HR 3.62, *p* < 0.001), need for mechanical ventilation (HR 2.17, *p* = 0.001), ≥ 2 comorbidities (HR 1.83, *p* = 0.049), SAH (HR: 1.56, *p* = 0.054) were predictors of mortality, as well as no use of prophylactic or therapeutic heparin (HR 3.60, *p* = 0.02). Multivariate analysis identified admission to the ICU (HR 8.98, *p* = 0.002) and advanced age (HR 3.37, *p* < 0.01) as independent predictors of mortality, although, use of heparin (HR 0.25, *p* = 0.001) was independently associated with a favorable outcome.

**Conclusion:**

This study confirmed the absence of a benefit associated with the use of HCQ in Brazilian patients hospitalized with COVID-19. However, prophylactic or therapeutic heparin was an independent predictor for reducing mortality in this population.

## Introduction

More than one year ago, in December 2019, an outbreak caused a respiratory illness in Wuhan, China, caused by a novel coronavirus (nCov) and later named Coronavirus disease 2019 (COVID-19) [1]. The severe form of this disease, characterized by hypoxemic respiratory failure, was called Severe Acute Respiratory Syndrome (SARS-Cov-2) [1]. Although we have made some progress on its therapy management, the disease has unfortunately resulted in 152.406.001 confirmed cases and 3.196.275 deaths globally till now [2]^.^Despite the fact that many potential vaccines were developed, there are immense logistical challenges in distributing to all vulnerable people, especially in developing countries. So, with the spread of COVID-19 moving on and a “third” wave emerging, there are no option out of caring for hospitalized patients with COVID-19.

Hydroxychloroquine (HCQ), an aminoquinolone used on the treatment of autoimmune diseases and malaria showed, on a small non-randomized trial, in the beginning of the COVID-19 pandemic, high efficacy with virologic cure when associated with azithromycin (AZTH) [3].

In Brazil, since March 2020, the Ministry of Health made available both Hydroxychloroquine and azithromycin for severe cases, at the discretion of the treating physician [4]. On May, both drugs had the recommendation expanded for mild cases [5]. With the first COVID-19 case recorded in Brazil located in São Paulo, considering the threat of the COVID-19 pandemic and with no randomized controlled trials available on timeframe, we performed an unicentric, prospective, interventional and consecutive non-randomized study to assess whether HCQ alone or in combination with azithromycin, would impact on mortality outcomes in hospitalized patients with documented pneumonia for COVID-19 on Public Health System in Brazil.

## Materials and methods

### Study design and patients

This is a prospective, non-randomized and unicentric cohort study conducted at Guilherme Álvaro Hospital (HGA) in Santos, São Paulo, Brazil. It is one of the largest hospitals in Metropolitan Region of Baixada Santista, 100% dedicated to Public Health System. Since March 2020, we have adapted isolation cohorts into COVID-19 dedicated beds as needs during the pandemic. The protocol was approved by the Committee for Ethics in Research (CEP number 3448). Furthermore, the study was registered at the National Research Ethics Commission (CONEP number: 30538920.9.0000.0008).

The study protocol was described for participants and written informed consents were obtained from all patients or their legal representatives. We included patients who were at least 18 years-old admitted to hospital with confirmed COVID-19 and radiographically documented pneumonia. An electrocardiogram was made at baseline, and as clinically indicated. Exclusion criteria was known hypersensitivity to HCQ, retinopathy, pregnancy or, in the physician’s view, any contraindication to the drug. Patients with exclusion criteria were used as control group and compared to those recruited to receive HCQ. For all patients a RT-PCR of the E, N and RdRP genes assay of nasal specimens, throat-swab specimens, or tracheal aspirate were used to confirm SARS-Cov-2 infection. Negative RT-PCR patients were submitted to a second RT-PCR test. Negative patients from both tests were excluded from the study, as well as patients only positive for serologic external results.

### Treatment regimens

After written informed consent provided by patients or by their legal representatives, eligible patients were recruited to HCQ group (alone or in combination with azithromycin) or to standard of care. Patients in the HCQ group received 400 mg hydroxychloroquine twice daily on first day followed by 400 mg for 4 days orally or via nasogastric tube in case of orotracheal intubation. Azithromycin 500 mg by oral, nasogastric or intravenous route for 5 days was allowed. The standard of care for COVID-19 was at the discretion of the treating physician. The use of corticosteroids and the antiviral oseltamivir was allowed, as well as other supportive care such as routine use of deep vein thrombosis prophylaxis and systemic antibiotics if bacterial infection coexisted. Specific protocols for the ventilatory management of respiratory failure and for sedation management throughout ICU stay was performed according to the hospital protocols.

### Outcomes

The primary end point was efficacy of hydroxychloroquine on death outcome. Secondary endpoints included analyses of any clinical or interventional parameters as predictive risk factors for death and lethality. All patients and their parameters were monitored until discharge.

### Laboratory and radiographic analysis

Routine blood examinations included basic metabolic panel, liver function, complete blood count and differential, ferritin, magnesium, C-reactive protein (CRP), lactate dehydrogenase (LDH), troponin and D-dimer. Baseline chest radiograph and/or computerized tomography (CT) were done at admission and furthermore as clinically determined by health care practitioners considering the individual evolution of each case. Laboratory assays for confirmation of SARS-Cov-2 were done as described above.

### Statistical analysis

Data are presented in accordance with the variables evaluated. Categorical variables are presented in absolute (N) and relative (percentage) numbers. Numerical variables are presented as mean and standard deviation (SD); as well as median, 25% percentile and 75% percentile, and minimum and maximum values. Overall Survival (OS) analysis was performed using the Kaplan–Meier (KM) method, and the Log-Rank (Mantel-Cox) test was used for comparisons between groups. Predictor analysis for the outcome was performed using Cox's semi-parametric univariate and multivariate regression or proportional hazards model, in which a value of p ≤ 0.15 was accepted for inclusion of the predictor in the final model. The order of inclusion of predictive factors in the multivariate analysis was performed using the lowest p value. The results are presented in Hazard Ratio (HR) and 95% Confidence Interval (95% CI). All analyzes were performed using the SPSS statistical software (IBM SPSS version 22.0) for Windows. A value of p ≤ 0.05 was assigned as significant.

## Results

### Clinical characteristics of the patients with COVID-19 infection

From March 28 to July 31, 2020, a total of 326 patients were hospitalized with suspected or external confirmed COVID-19 at our center. Of these 154 (47.2%) patients entered in this analysis. In this study, 172 (53.7%) patients were excluded because of negative internal RT-PCR for SARS-Cov-2, pregnancy or age < 18 years. A total of 95/154 (61.7%) received HCQ, and follow-up was completed on August 15, 2020. Baseline characteristics of patients are shown in Table 1. There was predominance of male gender (56.5%) and a median age of 60 years (range 21–90). Most patients, 136/154 (88.3%) had comorbidities, and the majority 51/154 (33.1%) had at least 2 comorbidities. Systemic arterial hypertension (SAH) was the most frequent comorbidity 78/154 (50.6%), followed by diabetes mellitus 50/154 (32.5%), cancer 32/154 (20.8%) and obesity 24/154 (15.2%). Dyspnea, cough and fever were the most common symptoms, with one of these occurring in 128/154 (83.1%). In 17/154 (11%) patients, atypical findings such as nausea, vomiting and diarrhea were present and 9/154 (5.8%) patients were asymptomatic or had no symptoms registered in medical records.

**Table 1:** Clinical characteristics, support and treatment of 154 patients with COVID-19 infection

COVID-19 patients	
Demographics	N (%)
Gender	
Male Female	87 (56.5) 67 (43.5)
Age (years)	60.0 (21.0 − 90.0)
Systemic Arterial Hypertension	
No Yes	76 (49.4) 78 (50.6)
Type 2 diabetes mellitus	
No Yes	104 (67.6) 50 (32.5)
Obesity	
No Yes	130 (84.4) 24 (15.6)
COPD	
No Yes	148 (96.1) 6 (3.9)
Asthma	
No Yes	149 (96.8) 5 (3.2)
Kidney disease	
No Yes	141 (91.6) 13 (8.4)
Hepatic disease	
No Yes	151 (98.1) 3 (1.9)
Cardiopathy	
No Yes	141 (91.6) 13 (8.4)
Cancer	
No Yes	122 (79.2) 32 (20.8)
Autoimmune disease	
No Yes	148 (96.1) 6 (3.9)
Dyslipidemia	
No Yes	147 (95.5) 7 (4.5)
Neurologic and psychiatric disorders	
No Yes	142 (92.2) 12 (7.8)
Smoking	
No Yes Ex	133 (86.4) 10 (6.5) 11 (7.1)
Alcoholic	
No Yes Ex	147(95.5) 3 (1.9) 4 (2.6)
Comorbidities	
No Yes	18 (11.7) 136 (88.3)
Respiratory symptoms	
No Yes	26 (16.9) 128 (83.1)
ICU admission	
No Yes	37 (24.0) 117 (76.0)
Oseltamivir	
No Yes	36 (23.4) 118 (76.6)
Azithromycin	
No Yes	24 (15.6) 130 (84.4)
Hydroxychloroquine	
No Yes	59 (38.3) 95 (61.7)
Corticosteroids	
No Yes	39 (25.3) 115 (74.7)
Heparin	
No Yes	8 (5.2) 146 (94.8)
DVT	
No Yes	152 (98.7) 2 (1.3)
Endotracheal Intubation	
No Yes	109 (70.8) 45 (29.2)
Pulmonary involvement > 50%	
No Yes No CT	58 (37.7) 59 (38.3) 37 (24.0)

*COPD* Chronic obstructive pulmonary disease, *ICU* Intensive care unit, *DVT* Deep vein thrombosis, *N* number of individuals, *%* percentage

Among all hospitalized patients, 117/154 (76%) were admitted on Intensive Care Unit (ICU) and 45/154 (29.2%) had endotracheal intubation (EOT) requirement (Table 1). Among patients submitted to lung computerized tomography, 59/117 (50.4%) had more than 50% involvement of the lungs. HCQ was given for 95/154 (61.7%) patients, and azithromycin for 130/154 (84.4%). In 118/154 (76.6%) patients, the antiviral oseltamivir was administrated and 115/154 (74.7%) received corticosteroids during the hyper-inflammation phase. Deep venous thromboembolism (DVT) was confirmed in 2/154 (1.3%) patients and heparin was used in 146/154 (94.8%) patients.

### Outcomes

**Fig. 1:**
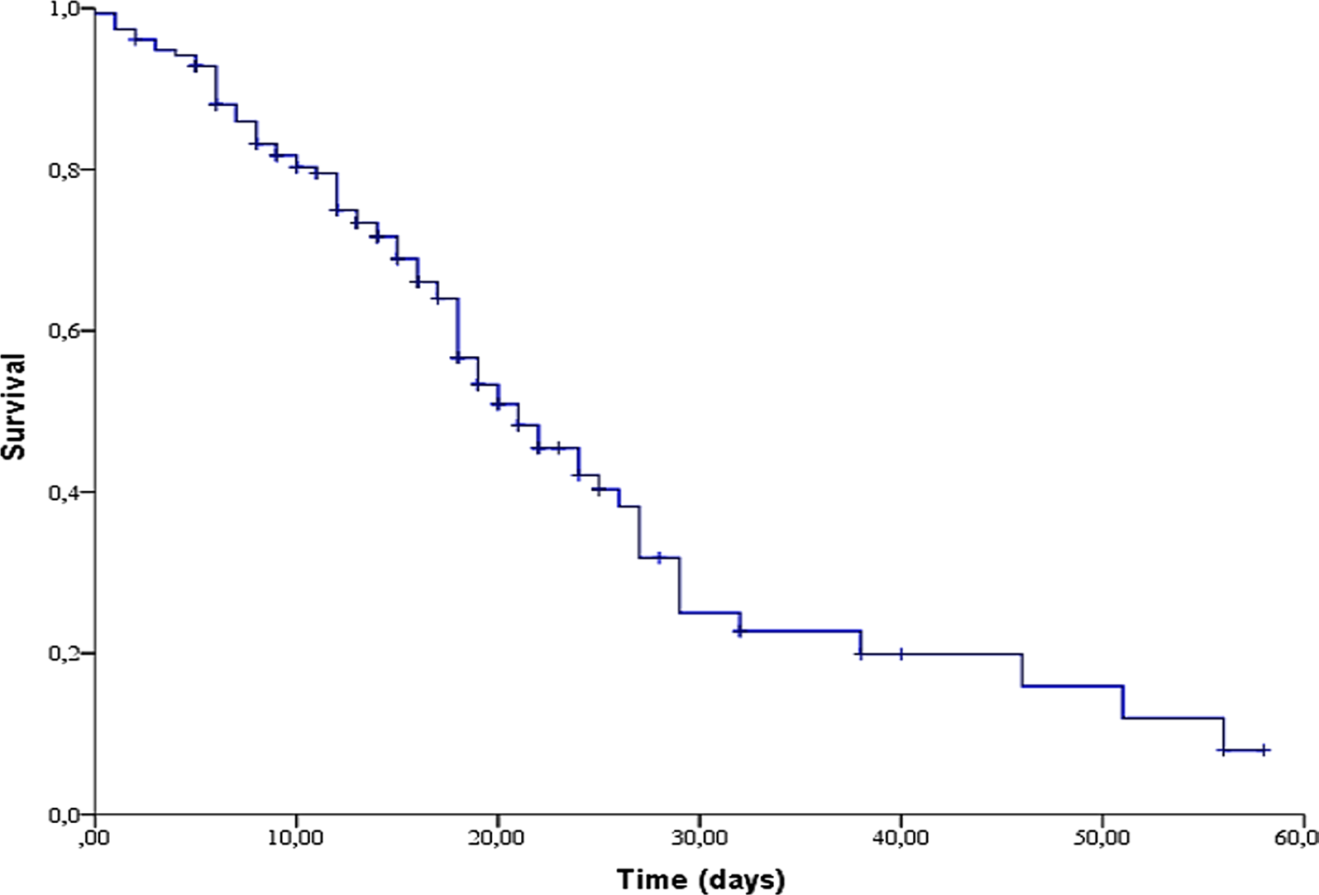
Overall survival of the 154 in-patients with COVID-19

In this cohort, the total death rate was 51.3% (79/154) and the median of overall survival (OS) from the onset of symptoms until the discharge from the hospital was 21 days (95% CI: 17.047 – 24.953) (Fig. 1).

**Fig. 2:**
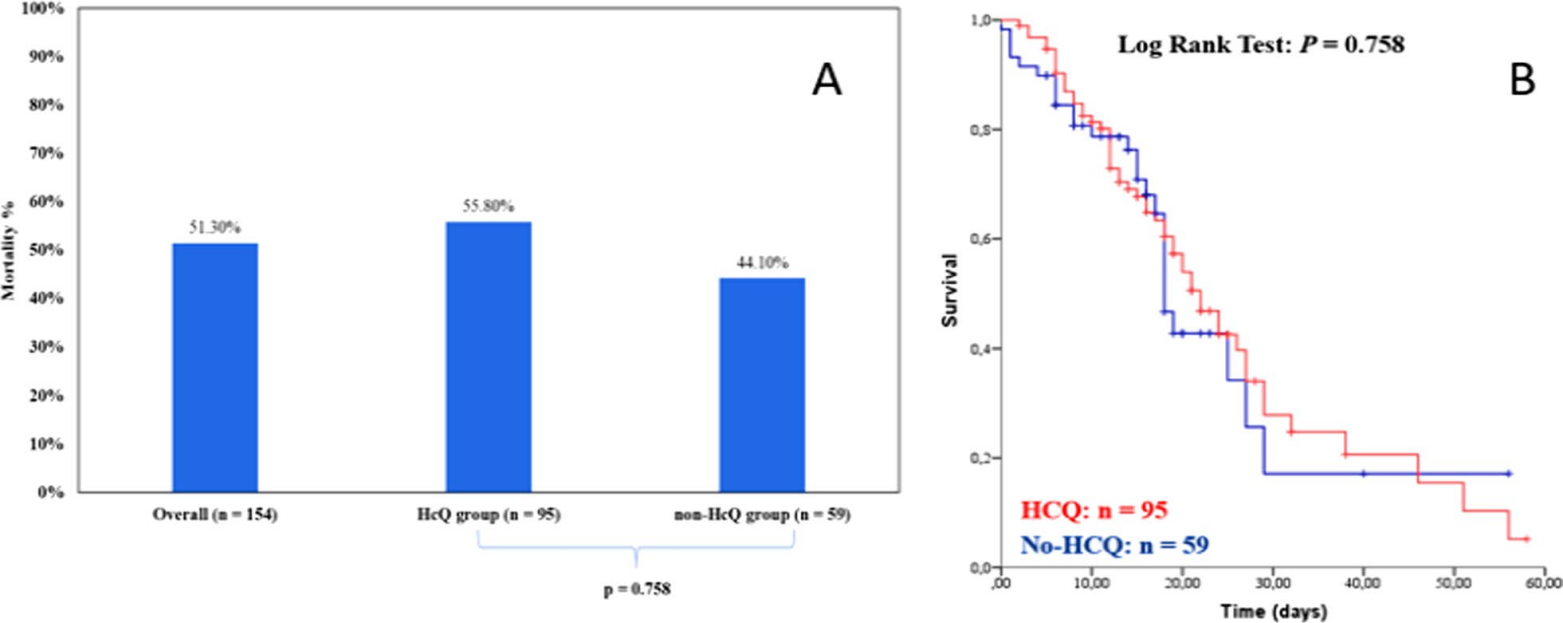
**A**—Mortality rates in COVID-19 hospitalized patients, groups HCQ and non-HCQ; **B**—Overall survival curve in the groups HCQ and non-HCQ. Comparison between groups by Log Rank Test (p = 0.758)

The primary end point of outcome, efficacy of HCQ on death is shown in Fig. 2. There were not significant differences of mortality among patients treated with HCQ 95/154 (61.7%) and those that did not received this drug 59/154 (38.3%). The mortality rate was 44.1% (26/59) in non-HCQ group and 55.8% (53/95) in HCQ group (median OS 22.0 days, [95% CI: 18.0–25.9] for HCQ group, and median OS 18.0 days, [95% CI: 16.4–19.5] for non-HCQ group, p = 0.758).

Table 2 shows a comparison of clinical and therapeutic characteristics between patients in the HCQ and non-HCQ groups included in our analysis.

**Table 2:** Comparison of clinical parameters between the non-HCQ and HCQ groups

COVID-19 patients (N = 154)				
Demographics	Non-HCQ N = 59 (%)	HCQ N = 95 (%)	p value	
Gender				
Male	36 (61.0)	51 (53.7)	0.372	
Female	26 (39.0)	44 (46.3)		
Age (years)	61 (22–89)	59 (21–90)	0.254	
Systemic Arterial Hypertension	30 (50.8)	48 (50.5)	0.969	
Type 2 diabetes mellitus	19 (32.2)	31 (32.6)	0.956	
Obesity	7 (11.9)	17 (17.9)	0.316	
COPD	2 (3.4)	4 (4.2)	0.798	
Asthma	0	5 (5.3)	0.073	
Kidney disease	6 (10.2)	7 (7.4)	0.543	
Hepatic disease	0	3 (3.2)	0.168	
Cardiopathy	6 (10.2)	7 (7.4)	0.543	
Cancer	20 (33.9)	12 (12.6)	0.002	
Autoimmune disease	2 (3.4)	4 (4.2)	0.798	
Dyslipidemia	4 (6.8)	3 (3.2)	0.294	
Neuro and psychiatric disorders	7 (11.9)	5 (5.3)	0.137	
Smoking				
Yes Ex	3 (5.1) 7 (11.9)	7 (7.4) 4 (4.2)	0.183	
Alcoholic				
Yes Ex	1 (1.7) 4 (6.8)	2 (2.1) 0	0.036	
Comorbidities	55 (93.2)	81 (85.3)	0.135	
Comorbidities ≥ 2	43 (78.2)	57 (70.4)	0.311	
Pulmonary involvement > 50%	20 (42.6)	39 (55.7)	0.163	
Pneumonia	55 (93.2)	94 (98.9)	0.071	
ICU admission	41 (69.5)	76 (80.0)	0.138	
Oseltamivir	33 (55.9)	85 (89.5)	< 0.001	
Azithromycin	39 (66.1)	91 (95.8)	< 0.001	
Corticosteroids	40 (67.8)	75 (78.9)	0.122	
Heparin	52 (88.1)	94 (98.9)	0.005	
Endotracheal Intubation	12 (20.3)	33 (34.7)	0.056	
DVT	1 (1.7)	1 (1.1)	1.000	

Comparison of categorical variables using the Chi-square test between non-HCQ and HCQ groups. *ICU* Intensive care unit, *DVT* Deep vein thrombosis, *N* number of individuals, *%* percentage

### Clinical factors associated with poor prognosis in patients with COVID-19

In univariate analysis, age ≥ 60 years was predictive for death (HR: 3.628; 95% CI: 2.208 − 5.960; *P* < 0.001) (Table 3). Gender, deep vein thrombosis and pulmonary involvement > 50% were not predictive for death. Systemic arterial hypertension presented a tendency for increment of death (HR: 1.564; 95% CI: 0.992– 2.466; *P* = 0.054). Single comorbidities as cardiovascular disease, obesity, chronic kidney disease (CKD), chronic obstructive pulmonary disease (COPD), asthma, DM, liver disease, cancer and smoking were not predictive for death. However, the presence of ≥ 2 comorbidities were statistically significant predictive of death (HR: 1.832; 95% CI: 1.003–3.346; *P* = 0.049). Gastrointestinal symptoms such as nausea, vomiting and diarrhea were not predictive of death (HR: 2.217; 95% CI: 0.893–5.507; *P* = 0.086), as well oseltamivir, HCQ, corticosteroids and azithromycin use. Heparin showed significant impact on survival, since patients who did not take heparin had a risk of death 3.6 times greater than those who used prophylactic or therapeutic heparin (HR: 3.606; 95% CI: 1.632 − 7.966; *P* = 0.002). Patients with necessity of mechanical ventilation also presented higher risk of death (HR: 2.176; 95% CI: 1.386–3.419; *P* = 0.001). Hospitalization at ICU (HR: 8.194; 95% CI: 2.001–33.553; *P* = 0.003) were also associated with high risk of death.

**Table 3:** Clinical risk factors associated with the death of patients with COVID-19

Clinical features	β-value	Hazard ratio	Confidence interval (95%)	*p *value
Gender	0.028	1.028	0.656–1.612	0.904
Age ≥ 60 years	1.289	3.628	2.208–5.960	< 0.001
Mechanical ventilation	0.778	2.176	1.386–3.419	0.001
Comorbidities	0.101	1.106	0.507–2.414	0.801
Comorbidities (≥ 2)	0.605	1.832	1.003–3.346	0.049
Heparin (no use)	1.282	3.606	1.632–7.966	0.002
Deep Venous thromboembolism	0.015	1.015	0.141–7.328	0.988
Pulmonary involvement > 50%	0.263	1.301	0.704–2.404	0.400
Hydroxychloroquine use	0.073	1.076	0.669–1.730	0.762
Asymptomatic	− 0.427	0.652	0.262–1.623	0.358
Gastrointestinal symptoms	0.796	2.217	0.893–5.507	0.086
Cardiovascular disease	0.563	1.757	0.871–3.544	0.116
Liver Disease	1.143	3.137	0.755–13.042	0.116
Chronic kidney disease	0.473	1.605	0.822–3.135	0.166
Asthma	− 3.058	0.047	0.001–19.973	0.322
COPD	0.349	1.418	0.444–4.530	0.556
Smoking	0.458	1.581	0.577–4.335	0.373
Diabetes	− 0.011	0.989	0.627–1.560	0.962
Obesity	0.061	1.063	0.618–1.826	0.826
Systemic arterial hypertension	0.447	1.564	0.992–2.466	0.054
Cancer	0.367	1.443	0.848–2.458	0.177
Oseltamivir	0.280	1.323	0.767–2.283	0.314
Corticosteroids	− 0.268	0.765	0.399–1.465	0.419
Azithromycin	0.125	1.133	0.596–2.156	0.703
Intensive Care Unit (ICU)	2.103	8.194	2.001–33.553	0.003

Univariate analysis for death in patients with COVID-19 by Cox Regression or Proportional Risk Model

In addition, the multivariate analysis showed four predictors factors of death in COVID-19 patients (Table 4). The ICU admission (HR: 8.980; 95% CI: 2.168–37.190; P = 0.002) was the main factor, followed by age above 60 years (HR: 3.475; 95% CI: 2.015–5.666; *P* < 0.001). In opposition, heparin use was protector of death (HR: 0.254; 95% CI: 0.113–0.569; *P* = 0.001). There was a tendency of protection against death for GI symptoms (HR: 0.404; 95% CI: 0.160–1.023; P = 0.056). Age above 60 years was also predictive for ICU admission (HR: 1.956; 95% CI: 1.328–2.882; *P* = 0.001) (Table 4).

**Table 4:** Association between clinical factors related to increased risk of death and increased risk of ICU admission in patients with COVID-19

Clinical features associated with death	β value	Hazard ratio	95% Confidence interval	*P *value
Age ≥ 61 years	1.218	3.379	2.015–5.666	< 0.001
Heparin (use)	− 1.372	0.254	0.113–0.569	0.001
Intensive Care Unit (ICU)	2.195	8.980	2.168–37.190	0.002
Gastrointestinal symptoms	− 0.905	0.404	0.160–1.023	0.056

Clinical features associated with ICU admission	β value	Hazard ratio	95% Confidence interval	*P *value
Age ≥ 61 years	0.599	1.820	1.245–2.660	0.002
Heparin (use)	− 0.776	0.460	0.200–1.059	0.068
Neurological diseases	0.777	2.029	0.965–4.263	0.062
Oseltamivir	− 0.363	0.695	0.446–1.085	0.110

Multivariate analysis for risk of death and for ICU admission in COVID-19 patients by Cox Regression or Proportional Risk Model

## Discussion

In this unicentric, prospective, interventional and non-randomized study, conducted in a public tertiary Hospital in Brazil, treatment with hydroxychloroquine had no impact on mortality outcomes in hospitalized patients with documented pneumonia for COVID-19. When we compared the mortality rate among patients treated or not treated with HCQ we did not find any significant difference (55.8 vs 44.1%; *P* = 0.758).

At the beginning of a frightening and unique pandemic moment, with no randomized controlled trial available, with local recommendations in favor of the use of the HCQ, our study was carried out based on ethical concerns and in order to answer the question of its benefit and guide us towards of good quality for clinical decisions. Also, the rationale and decision for prescribing this drug was based on the preliminary study in France, which showed virological cure in patients who received HCQ combined with azithromycin, the confidence and experience of Brazilians physicians with the use of HCQ for decades on the treatment of malaria, autoimmune disorders and prompt availability of the drug [2, 3].

With limitations regarding an unicentric, small number of cases and a more serious manifestation of SARS-Cov-2 infection in our cohort, our finding is consistent with a more robust data showing no benefit of HCQ in adults hospitalized with COVID-19 [5, 6]. Although in our study the cardiac toxicity effect of HCQ could not be unreliable assessed because most patients were using azithromycin and synergistic toxic effects are well known, the 5 days use of both drugs were done inpatient, so that monitoring and adherence were well controlled [7]. Also, another limitation of our study was the lack of a panel flu-test. Although part of our study occurred during influenza season and oseltamivir was administered for 76.6% of the patients, clinical symptoms can overlap with the COVID-19 [8]. Yet, RT-PCR for SARS-Cov-2 was positive in all cohort.

In our cohort, 51.3% (79/154) evolved to death during hospitalization in a median time of 21 days (95% CI, 17.047–24.953) from the onset of symptoms. In agreement with others reports, the most frequent symptoms of our patients with COVID-19 were dyspnea, cough and fever, found in 128/154 (83.1%) patients [9, 10]. Likewise, unspecific gastrointestinal symptoms of nausea, vomiting and diarrhea were seen in 17/154 (11%) patients. As expected, respiratory symptoms were present in the majority of patients.

In comparison to others studies, our casuistic comprehended more vulnerable patients with higher risk to develop complications and death by COVID-19. In our cohort, the median age of patients were 60 years and 136/154 (88.3%) of them presented comorbidities, being systemic arterial hypertension the most frequent, observed in 50.6% of them (78/154). Malignant neoplasms were present in 20.8% (32/154) of patients. Therefore, these worse clinical aspects of our casuistic may justify our higher mortality in comparison with other studies. Fei Zhou et cols. reported that in a total of 191 inpatients in a Hospital in Wuhan, China, the epicenter of the beginning of the SARS-Cov-2 pandemic, 137 were discharged and 54 (28.3%) died [11]. In this report 91 (48%) of patients presented comorbidities. In multivariate analysis, they showed, like us, that older age was associated with higher risk of in-hospital death. [11]. Wu Z et al., showed that 49% of inpatients with severe COVID-19 had comorbidities [12].

The univariate analysis showed that age equal or higher than 60 years, mechanical ventilation necessity, presence of at least two comorbidities, no heparin use and SAH were predictive of death. However, in multivariate analysis age equal or higher than 60 years and EOT necessity remained as independent factors for mortality. Interestingly, heparin use was beneficial and related to lower mortality. Age equal or higher than 60 years was also predictive for ICU admission in multivariate analysis. The ICU care was required for 76.9% (117/152) patients and 67.5% (79–117) of them evolved to death. During this study, the ICU beds were widely available and all patients were immediately admitted in this unit when necessary. In the beginning of the pandemic it was verified that risk of death increased with older age. In a Chinese series, the mortality was 0.5% in patients younger than 50 years, 2% (50–59 years), 4% (60–69 years), 8% (70–79 years) and 16% (> 80 years) [13]. The authors reported 67% of mortality in patients over 80 years, 20% between 70 and 79 years and 8% between 60 and 69 years [13]. However, the comparison of mortality among many countries is not all reliable, because of different medical resources.

Despite of the scientific advances acquired last year, till now some parts of the world are astonishing and in some words lost with this disease. In fact, in the beginning old people were dragged into this tourbillion named SARS-Cov-2 pandemic and although our cohort also found age an important and independent factor for mortality in patients with COVID-19 hospitalized, now we are faced with new mutations, younger patients requiring medical assistance and dealing with the hospitalized patient is an everyday practice for many physicians. Unfortunately, in Brazil, apart from initial recommendations regarding HCQ use, no former protocol regarding the best of care for inpatient COVID-19 was made available by the Ministry of Health until April 2021.

Aging is associated with decline of immune function, T and B lymphocytes dysfunction [14–16] as well as lower level of interferon type 1 [17–19]. On the other hand, old people carry more comorbidities and consequently higher probability to develop more complication in the setting of infection. Aging is also associated with a higher inflammatory steady-state caused by a higher production of cytokines [20]. These conditions certainly have contributed to the clinical severity and higher mortality associated with COVID-19 in older people [20].

Another important aspect of the COVID-19 is that an explosion of thrombi-inflammation seen in the hyper inflammation phase results in micro-thrombosis of lung vasculature and in a severe interstitial pneumonia characterized by severe hypoxemia with acute respiratory insufficiency, with necessity of high concentration of oxygen supplying and, in more severe patients, invasive ventilatory support. Less frequently, patients can also develop acute renal failure increasing the gravity of the disease [21].

Because of this physiopathology and the high rate of thromboembolic complication in patients with COVID-19 [22], prophylactic administration of low-molecular weight heparin (LMWH) for hospitalized COVID-19 patients is recommended [23]. However, Paranjpe et al. showed that systemic anticoagulation has been associated with COVID-19 benefit only in subgroup of patients with EOT [24]. Unexpectedly, at the time of this study was conducted, in the beginning of the pandemic in Brazil, we found that heparin was and independent protector factor against mortality in multivariate analysis in our casuistic. Beyond it anticoagulation action, heparin also have others functions, like inhibition of heparase enzyme and linked to endothelial leakage. It also capable of neutralizing chemokines, cytokines and the cell influx of extracellular cytotoxic histones. Moreover, heparin prevents the viral cellular uptake and interfere with leukocyte trafficking [23, 25], directly interfering with pathophysiological mechanisms associated with viral infection progression and endothelial damage.

Some limitations should be highlighted in our study. Although evaluation by computed tomography is considered the gold standard for assessing pulmonary parenchyma involvement by COVID-19 and quantifying its extension, only 76% of our patients (117/154) were evaluated using this imaging technique. Most of the remaining cases had an image evaluation done by chest radiography, because these are severe cases, including patients on mechanical ventilation, whose transport to the imaging sector offered logistical difficulties and imminent risk to the patient's life during transport. However, these cases showed evident involvement of the pulmonary parenchyma with more than 50% involvement of this organ on the chest X-ray.

Individuals with COVID-19 included in the HCQ and non-HCQ groups have comparable clinical characteristics as summarized in Table 3, with the exception of a higher percentage of neoplasia and alcoholism in the non-HCQ group. Regarding therapeutic management variables, the non-HCQ group had a lower percentage of exposure to oseltamivir, azithromycin and heparin, with a statistically significant difference. In multivariate analysis, therapy with oseltamivir and azithromycin had no impact on risk of death.

Unlike what was demonstrated by the RECOVERY study, where exposure to low doses of corticosteroids (dexamethasone) was associated with a 28-day mortality reduction in patients with COVID-19 who required mechanical ventilation [6], our study did not demonstrate a statistically significant association between treatment with corticosteroids and reduced mortality in the population included. We believe that our results may have been influenced by the lack of standardization regarding the type, dose and timing of corticosteroid introduction.

With regard to the use of heparin, only 5.2% of the patients (8/154) were not exposed to this drug, which makes the comparison groups discrepant and may lead to an analysis bias. The low prevalence of individuals in the group not exposed to heparin may affect the conclusions drawn from the results found in our study. However, the analysis of hazard ratio (HR) contemplates this difference, which makes the results found in our cohort statistically acceptable. Among the 8 patients not exposed to heparin, 5/8 did not receive the medication because they were severely thrombocytopenic and/or with active bleeding and 2/8 evolved with cardio-respiratory arrest in the first 24 h of hospital admission, making it impossible to prescribe the medication. This may have influenced the results found, since patients not treated with heparin may have had a higher mortality due to present a more severe disease. However, one point that corroborates the potential benefit of heparin in patients hospitalized with COVID-19 was the low incidence of thromboembolic events (1.29%—2/154) observed in our cohort, and possibly, this is a reflection of the massive use of prophylactic heparin in the patients included in the study. Although there was no active search for thromboembolism, several case series demonstrate higher rates of thromboembolic events in populations similar to ours [22, 26].

Despite of this work has been done to answer a question about HCQ in the setting of the COVID-19, it allowed us to take a picture of the clinical characteristics of our patients in the setting of this disease. We also could identify the main factors associated with mortality in our center.

In conclusion we confirmed that age and association of comorbid were predictive of poor prognosis in COVID-19 and higher chance of mortality. However, heparin administration was associated with less mortality and hydroxychloroquine did not influence the mortality.

## Data Availability

All data generated and analysed during this study are included in this published article. The raw data for this study are in the possession of the correspondence author and may be made fully available in the event of a request to the correspondence author via e-mail.
